# Soft sensor based on 2D‐fluorescence and process data enabling real‐time estimation of biomass in *Escherichia coli* cultivations

**DOI:** 10.1002/elsc.201900076

**Published:** 2019-11-11

**Authors:** Benjamin Bayer, Moritz von Stosch, Michael Melcher, Mark Duerkop, Gerald Striedner

**Affiliations:** ^1^ Department of Biotechnology University of Natural Resources and Life Sciences Vienna Austria; ^2^ School of Chemical Engineering and Advanced Materials Newcastle University Newcastle upon Tyne United Kingdom; ^3^ Institute of Applied Statistics and Computing University of Natural Resources and Life Sciences Vienna Austria; ^4^ Austrian Centre of Industrial Biotechnology Graz Austria; ^5^ Novasign GmbH Vienna Austria

**Keywords:** bioprocess engineering, chemometric modeling, multivariate adaptive regression spline, process monitoring, Quality by Design

## Abstract

In bioprocesses, specific process responses such as the biomass cannot typically be measured directly on‐line, since analytical sampling is associated with unavoidable time delays. Accessing those responses in real‐time is essential for Quality by Design and process analytical technology concepts. Soft sensors overcome these limitations by indirectly measuring the variables of interest using a previously derived model and actual process data in real time. In this study, a biomass soft sensor based on 2D‐fluorescence data and process data, was developed for a comprehensive study with a 20‐L experimental design, for *Escherichia coli* fed‐batch cultivations. A multivariate adaptive regression splines algorithm was applied to 2D‐fluorescence spectra and process data, to estimate the biomass concentration at any time during the process. Prediction errors of 4.9% (0.99 g/L) for validation and 3.8% (0.69 g/L) for new data (external validation), were obtained. Using principal component and parallel factor analyses on the 2D‐fluorescence data, two potential chemical compounds were identified and directly linked to cell metabolism. The same wavelength pairs were also important predictors for the regression‐model performance. Overall, the proposed soft sensor is a valuable tool for monitoring the process performance on‐line, enabling Quality by Design.

**Abbreviations**
5x‐CV: five‐fold cross‐validation
CPP: critical process parameter
DoE: design of experiments
ex/em: excitation/emission
LoBo‐CV: leave‐one‐batch‐out cross‐validation
MARS: multivariate adaptive regression splines
PARAFAC: parallel factor analysis
PAT: process analytical technology
PC: principal component
PCA: principal component analysis
QbD: Quality by Design
RMSE: root mean squared error
SGR: specific growth rate
VIP: importance of the input variables

## 1 INTRODUCTION

### 1.1 Recombinant protein production

At present, operators try to ensure process performance consistency by operating the process according to a fixed protocol, with deviations leading to post‐process investigations. However, process inputs succumb inevitably to variability, and the quality is examined only at the end of the process. At this point, it is determined whether the outputs meet the required standards or whether the batch must be withdrawn 1. Narrowing the output specifications to guarantee higher quality standards results in increasing numbers of rejected batches. This consequently leads to an enormous loss of energy, time, money, and goods 2. Another point to consider is that the application of fixed process settings gives rise to variable outputs. This can be troublesome if a certain biomass is needed for a specific process operation, for example, induction in *Escherichia coli*. Thus, it is of great interest to know the current biomass concentration at any time during the process.

### 1.2 Quality by design and process analytical technology

Pharmaceutical manufacturing is tightly controlled by the authorities. The current procedure, namely Quality by Testing, is disadvantageous from an economic aspect and is associated with long development times. To tackle these batch‐to‐batch variations and inconsistencies and to increase process understanding, the FDA published the process analytical technology (PAT) guidance for the biopharmaceutical industry in 2004. This guidance proposes the use of risk assessments for the identification of critical process parameters (CPPs), whose impact on the product's critical quality attributes (CQAs) should be studied during process development 3. Typically, this is accomplished utilizing design of experiments (DoE) approaches to analyze the CPPs’ multidimensional impacts on the CQAs. Subsequently, a monitoring strategy must be defined to ensure that the process performs as expected and to provide an opportunity to counteract any input variations that may occur. This allows for more robust and uniform outputs with respect to quality assurance and proper risk management 4.

The gathered process knowledge should be used to switch from a Quality by Testing to a Quality by Design (QbD) approach 5. This will lead to a well‐understood process to the extent that the monitored variables and the quality are guaranteed by the process itself. Although plenty of information about QbD and its application is already available, QbD is still far from being implemented as the new state of the art, in particular for upstream bioprocess operations 6, due to the lack of appropriate monitoring tools.

### 1.3 Advanced on‐line sensor systems and soft sensors

Progress has been seen regarding on‐line monitoring tools for the PAT concept, not only for microbial but also for mammalian cell cultures. Many optical sensor systems using different spectroscopic techniques are currently used in the industry 7. For instance, simple in situ microscopic techniques are already in use 8, as well as more advanced Raman spectroscopy 9 or infrared spectroscopy 10 techniques. Fluorescence spectroscopy techniques are also associated with the group of advanced sensors. Fluorescence spectroscopy is based on determining the specific excitation and emission wavelengths of a compound in order to identify it qualitatively and quantitatively, in the range of the measured 2D‐fluorescence spectrum 11, 12. This sensor type, together with other spectroscopic methods, is suitable for on‐line applications, since continuous, non‐invasive, and nondestructive measurements are possible and no sample needs to be drawn, thereby eliminating the risk of contamination. In addition, the determination of various compounds within a single measurement renders these techniques fast and robust, as well as cost‐efficient. 2D‐fluorescence spectroscopy is very sensitive and allows fluorescing molecules to be monitored inside and outside the cell. This technique has already been used to monitor microbial cultures and has been shown to reveal information about the physiological status of the cells 13.

**PRACTICAL APPLICATION**

We propose a workflow to establish a soft sensor with an exceptional generalization capacity and wide applicability. The presented soft sensor is able to accurately estimate biomass concentrations on‐line. Therefore, no analytical time delay occurs. This is of great interest to manufacturers, for monitoring and controlling their processes. For example, using this soft sensor, the induction could be always initiated at a defined biomass concentration. Moreover, the described modeling algorithm lists the predictive importance of all possible model parameters, enabling process understanding under the QbD concept. Furthermore, the soft sensor performance was tested by applying it to fermentations with different parameter settings as used for the design space characterization (up to three altered parameters). Despite the new fermentation settings, accurate estimations were obtained, which demonstrates the ability of the soft sensor to monitor the biomass concentration of different processes in real time.

Changes in the on‐line signals, (e.g., fluorescence) can be used for chemometric modeling, to build so‐called soft sensors for estimating various bioprocess quality attributes or variables of interest in real time. In particular, multivariate data analysis (MVDA) is used to investigate the correlations between on‐line and off‐line measurements. With the help of machine learning methods, these on‐line signals can be translated into the corresponding off‐line variables 14. Hence, it is possible to estimate and monitor specific complex variables via unspecific on‐line signals in real time and moreover, to estimate non‐fluorescent substances via their stoichiometric relationship to fluorescent compounds within the process 15.

### 1.4 Multivariate data analysis and regression models

Unsupervised methods for exploratory data analysis, for example, parallel factor analysis (PARAFAC) 16 or principal component analysis (PCA) 17, are applied to gain deeper knowledge and to reveal information hidden within the data. In this way, important chemical compounds can be identified and further insights into the physiology of the cell can be obtained. An effective way of extracting information from process data and building soft sensors exploiting the hardware sensors used is MVDA 18. These types of soft sensors are based on data and do not necessarily need further knowledge or mechanistic understanding. Some frequently applied machine learning methods make use of partial least squares regression, but non‐linear methods such as random forest, artificial neural networks and support vector machines are also in use 19. A powerful approach that takes strong interactions between variables into account and is also able to model non‐linearities, is the multivariate adaptive regression spline (MARS). Due to its dynamic adaptability in selecting subsets of local variables, this algorithm can be seen as an ideal candidate for process modeling. The MARS algorithm has not been used for soft sensor‐building in upstream processes to date. MARS is considered as an extension of linear models and is well suited to dealing with high dimensional input data 20.

Data preprocessing should also be taken into consideration before model‐building, in order to develop a more robust model, for example, by using the z‐score, that is, autoscaling 21. This enables more accurate comparability between different processes by contemplating only the change over time instead of the quantity of the measured units 22. The common way to validate the developed model is to apply the model to an independent test set, also referred to as an external validation set, which has not been used for model training.

This work presents a new soft sensor based on 2D‐fluorescence data and other on‐line process data. MARS was used for model‐building, due to its simplicity compared to other algorithms that can deal with a large number of input variables, multi‐collinearity and non‐linearity. The soft sensor performance for on‐line monitoring of the biomass is assessed for the 27 distinct experiments of a complete DoE study, as well as for two DoE‐independent test runs. Exploratory data analysis was performed to gain insight into the data and to investigate the fluorescence spectra. The important wavelengths for biomass sensing and the potential underlying chemical compounds accounting for cell metabolism, were identified and described in detail using PCA and PARAFAC.

## 2 MATERIALS AND METHODS

### 2.1 Process conditions

*E. coli* (HMS174 (DE3)) was cultivated in fed‐batch fermentations at a 20‐L scale, expressing recombinant human Cu/Zn superoxide dismutase. All details of the bacterial strain, plasmid, cultivation, induction conditions, and on‐line and off‐line monitoring, have already been described elsewhere 23, 24. The impact of three CPPs on the process performance using DoE, was studied. These were temperature (30, 34, and 37°C), the induction ratio (0.2, 0.5, and 0.9 µmol IPTG/g cell dry mass) and the specific growth rate (SGR) (0.10, 0.15, and 0.20 hours (h)^−1^). The SGR was held constant by an exponential substrate feed. All corresponding reactor volumes of the fed‐batch fermentations are provided in Supporting Information Figure S1. This resulted in a 3‐D design space with 27 CPP combinations. This design extends the space investigated in the earlier study.

### 2.2 Data set

The data set consisted of 33 fermentations, with 27 experiments from the DoE study, together with two duplicates and one quintuplicate. Furthermore, two differing CPP settings, still located in the investigated space, were used as a test set. All CPP settings within the design space are listed in Supporting Information Table S1. The biomass (target variable) was measured once prior to the induction and thereafter at hourly intervals, via thermogravimetric analysis. The five variables available on‐line (accumulated feed in grams, base in grams, inductor in µmol, temperature in°C and inlet air in standard liters per minute), as well as the 120 excitation/emission (ex/em) wavelength pairs measured by a 2D‐fluorescence probe (BioView^®^, Delta Light & Optics, Denmark), were utilized as input data for model‐building. The inlet air and the stirrer speed (not used for model‐building), were used to keep the dissolved oxygen set point at 30% during the fermentations. The 2D‐fluorescence probe measured the cultivation broth ranging from ex270/em310 up to ex550/em590, in 20‐nm steps.

Exploratory data analysis and soft sensor development were performed using MATLAB (2016b, MathWorks, USA), together with the three freely available packages ARESLab [25], N‐way 26, and drEEM 27. A graphical overview of the complete development process for the soft sensor, from the data gathering stage to until the final model, is provided in Supporting Information Figure S2.

### 2.3 Data preprocessing

#### 2.3.1 Standardization of the fluorescence data

To take the change in the measured spectra into account, rather than the absolute quantity, the 120 ex/em pairs were standardized along the time domain. This was done for each observation prior to modeling using the MATLAB function *zscore*.

#### 2.3.2 Time alignment

The on‐line data set used for training (values available every 3 min), consisted of 125 variables and 11126 observations and was time‐aligned to the respective sampling points of the single target variable (values available every hour), consisting of 690 observations (12 to 25 per fermentation).

### 2.4 Exploratory data analysis of the fluorescence data using PCA and PARAFAC

PCA and PARAFAC, as described by Bro 28, were used on the complete fluorescence data set to gain more specific insights into the data and the underlying structures. First, a PCA was performed on the fluorescence data, to unveil the latent structures that explain most of the variance in the data. To determine the location of the underlying fluorescent compounds in the spectrum, PARAFAC was also applied. PARAFAC, unlike PCA, decomposes the fluorescence matrices not only into scores and loadings but also into a third dimension, resulting in three different modes. In the case of the fluorescence data, the first mode represents the sample and is directly proportional to its concentration. The second mode represents the excitation and the third mode represents the emission wavelength of the respective analyte. By joining the second and third modes, the location of the respective factor in the 2D‐fluorescence spectrum is displayed. Thus, PARAFAC overcomes the rotational freedom of PCA, making it a better choice for the analysis of fluorescence spectra.

### 2.5 Model development

For model training, all fermentations were used. In total, three different models were developed: one using the five available on‐line process variables mentioned above, one with only the fluorescence data, and one with both types of data merged. The best input to the model with respect to accurate biomass estimation for internal validation, was used as the final model. The established single‐response models were based on the MARS algorithm. This algorithm is well suited to regression modeling of high‐dimensional data. It is flexible and based on the expansion of spline basis functions as described by 29. The model‐building comprises two phases, the forward selection followed by the backward deletion of input variables. Detailed information about the MARS algorithm, the workflow for building the MARS model, the basic functions included in the final model and exemplary trajectories of the used inputs, are provided in the Supporting Information Figure S3.

#### 2.5.1 Relative input variable importance

The importance of the input variables (VIP) was assessed for subsequent use. The VIP is defined by the square root of the generalized cross‐validation of the MARS model excluding that variable (still including all basis functions), minus the square root of the corresponding full MARS model's generalized cross‐validation. For ease of interpretation, all relative VIPs were scaled in such a way that the most important variable possessed a value of 100.

#### 2.5.2 Model performance criteria

To guarantee that the developed models possess optimal generalization capabilities, various performance criteria were considered. To validate the models, the root mean squared error (RMSE) (Eq. (1)) and the percentage model error (Eq. (2)) were computed, together with the number of observations (*N*), the respective value of the biomass concentration (*y*),the index (i = 1:N) and its estimated counterpart (*ŷ*).

(1) $$RMSE\;=\;\sqrt{\frac{1}{N}*\sum {\left(y_{\left(i\right)}-{\hat{y}}_{\left(i\right)}\right)}^{2}}$$

(2) $$Error\;\;\left[\%\right]=\;\frac{100}{N}\;*\;\sum \frac{\left|y_{\left(i\right)}-{\hat{y}}_{\left(i\right)}\right|}{y_{\left(i\right)}}\;$$

The SD in Eq. (3) was calculated using the measured value (*y*), the mean value (*y*_mean_) and the number of observations (*N*) for each time point (*t)*.

(3) $$S\;D_{\left(t\right)}=\sqrt{\frac{1}{N}*\sum {\left(y_{\left(t\right)}-{y_{\mathrm{mean}}}_{\left(t\right)}\right)}^{2}}$$

The confidence band is provided by calculating the upper and lower 95% confidence interval (CI) in Eq. (4) for each value (*y*) and the respective SD for each time point (*t)*.

(4) $$95\;\%\;C\;I_{\left(t\right)}=\;y_{\left(t\right)}\pm 1.96\;\;\times \;SD_{\left(t\right)}$$

#### 2.5.3 Model validation

Two internal validations were performed. The five‐fold cross‐validation (5x‐CV) in which a random 20% of the data were not considered for the model‐building, was used to test the performance. This procedure was repeated four more times until every observation was used for model validation. The second validation used the leave‐one‐batch‐out cross‐validation (LoBo‐CV) method. For the LoBo‐CV, there was always a complete fermentation that was not considered for training, and the model, which was built on all the other fermentations was validated on this particular fermentation. Again, this procedure was repeated until each fermentation had been used once as a validation set. The performance of the three established models regarding the internal validation was used as the quality criterion for choosing the best input for the final model. The final model was applied to a test set (external validation) to investigate how it performed on new data. The external validation consisted of the two different fermentation settings, as described previously, which had not been used for validation.

## 3 RESULTS

### 3.1 Experimental biomass results

**Figure 1:**
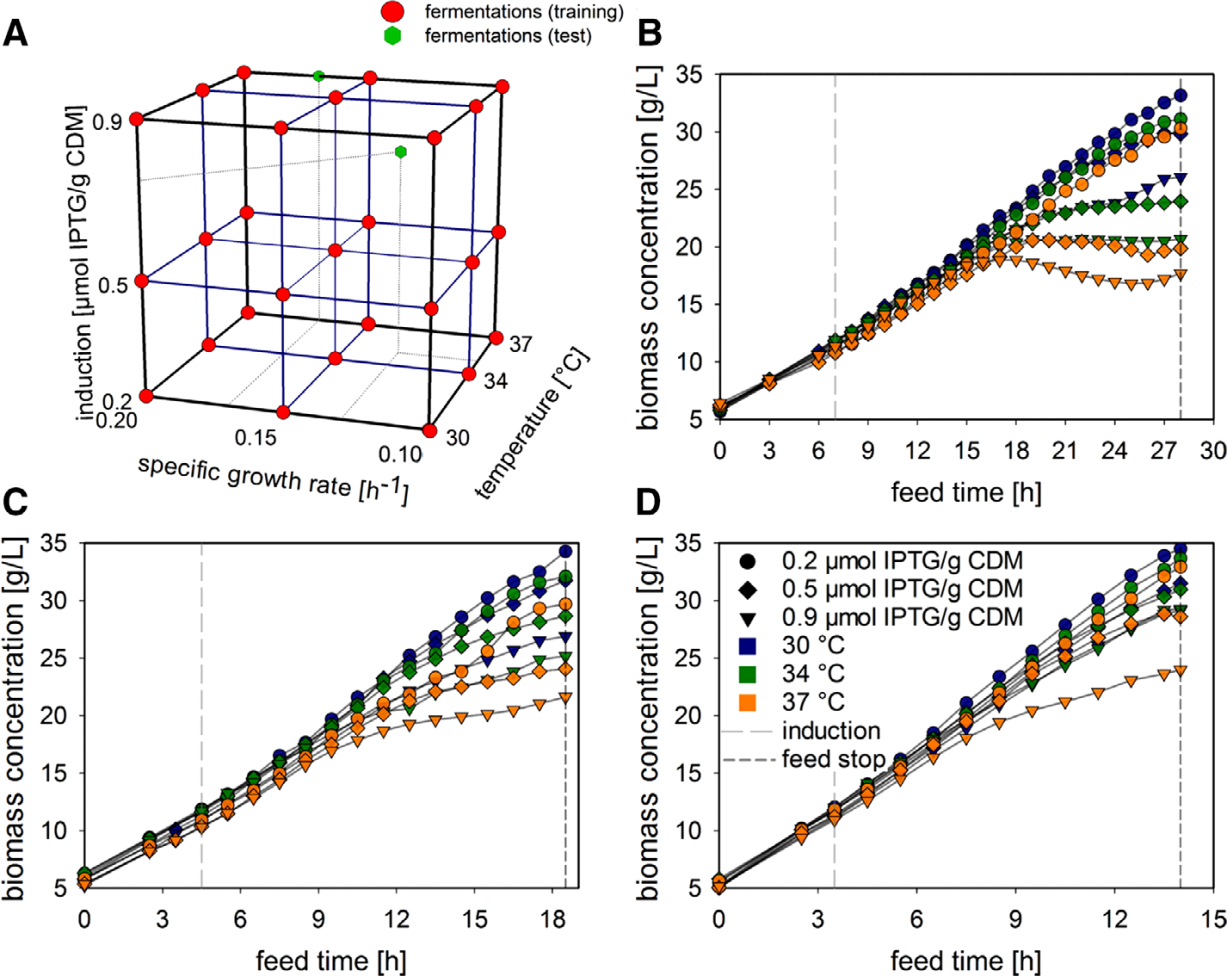
Experimental biomass results of the investigated design space. (A) DoE for the three factors (red), and test fermentations (green). (B–D): Biomass trends as a function of the SGR for slow (B), medium (C) and fast (D) growth. The induction ratios are presented with different symbols, that is, 0.2 (dot), 0.5 (square), and 0.9 (triangle), while the varying temperatures are displayed in different colors (30°C in blue, 34°C in green, and 37°C in orange)

Biomass trends of the 27 characterized DoE points (Figure 1A) are presented, separated into the three SGRs (µ = 0.10, µ = 0.15, and µ = 0.20) (Figure 1B–D). The biomass concentration trajectory shows the variation as a function of each CPP combination, providing an insight into the challenge presented to the soft sensor. The respective time points of induction (after one doubling time) and the feed stop (after four doubling times in total), are given.

A distinctive tendency towards higher biomass concentrations is visibly associated with lower induction and lower temperature settings, which were uniform for all SGR settings. For µ = 0.10, the maximum difference between the settings is 15.5 g/L (Figure 1B, ranging from 17.7 to 33.2 g/L). An increase in temperature or induction subsequently causes lower biomass concentrations over the whole fermentation. This effect is diminished by increasing the SGR. For µ = 0.15, the maximum difference is 12.7 g/L (Figure 1C, 21.6 to 34.3 g/L) and for µ = 0.20 it is only 10.5 g/L (Figure 1D, 24 to 34.5 g/L). The CPP combinations for the test set were also located in regions where high impacts on the biomass are reported. Therefore, it can be assumed that they will be quite challenging for the soft sensor to estimate, producing a suitable quality criterion for external validation.

### 3.2 Exploratory data analysis of the 2D‐fluorescence spectra

**Figure 2:**
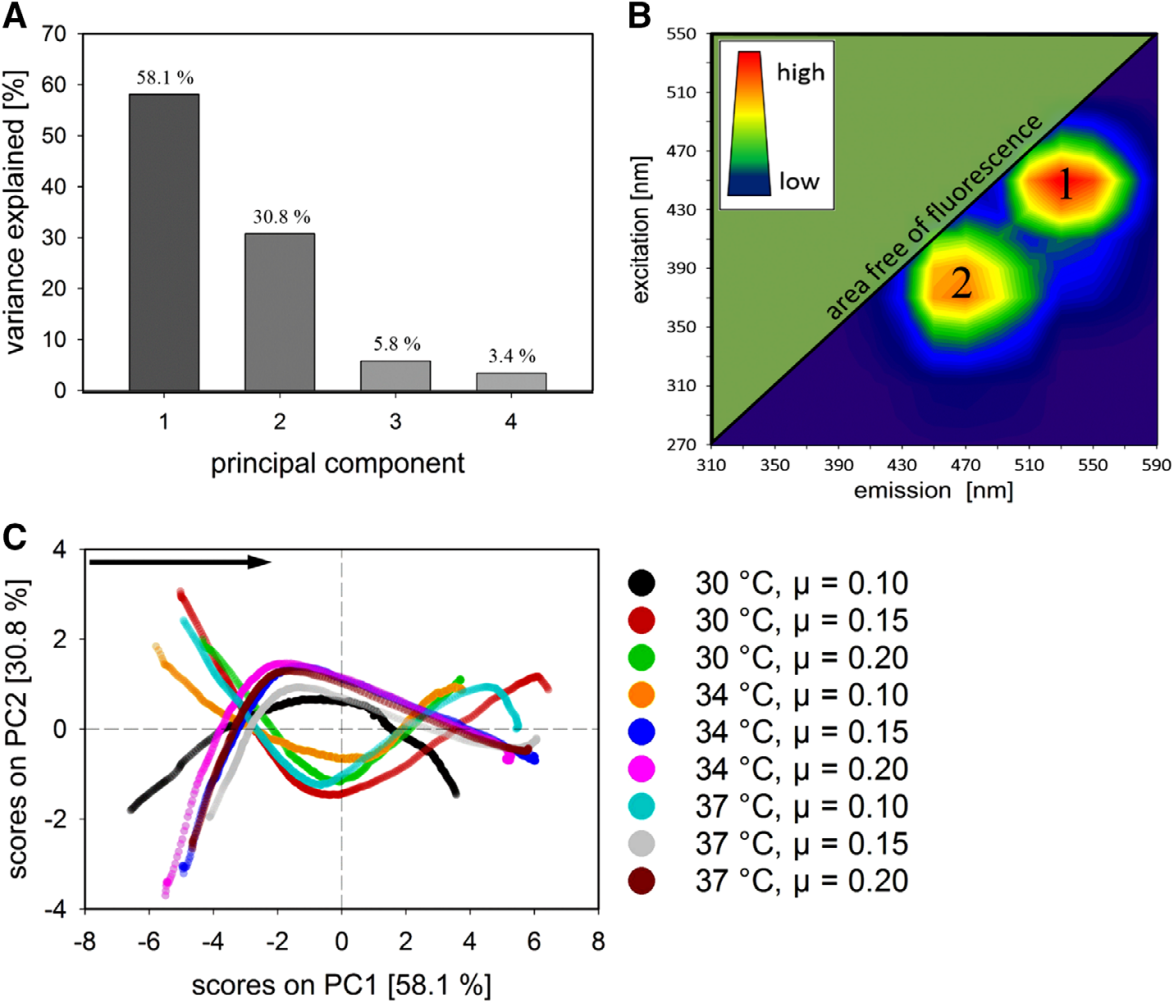
Results of the exploratory data analysis of the 2D‐fluorescence spectra. (A) Variance explained by principal components for the fluorescence spectra. (B) The location of the compounds ex450/em530 (factor 1) and ex370/em470 (factor 2) determined using PARAFAC in the 2D‐fluorescence matrix. The fluorescence‐free area and the color scale representing the intensity from dark blue (lowest value) to red (highest value), are shown. (C) Scatter plot of the scores for PC 1 versus PC 2 for all CPP combinations carried out at an induction ratio of 0.9. The direction (black arrow) and the different CPP settings (color scale) are indicated

To gain deeper process understanding, unsupervised learning was performed, and the measured data derived from the advanced on‐line probe were inspected. A PCA of the 2D‐fluorescence data set revealed that three to four principal components (PC) describe almost all of the variance in the data. These are PC 1 (58.1%), PC 2 (30.8%), PC 3 (5.8%), and PC 4 (3.4%), as shown in Figure 2A. To determine the ex/em pairs that are accountable for the changes in the spectrum, PARAFAC was performed on the fluorescence data. Two main factors were identified in the 2D‐fluorescence spectrum, as shown in Figure 2B, namely, ex450/em530 (factor 1) and ex370/em470 (factor 2). These factors correspond to underlying fluorescent chemical compounds inside the cell and the broth, which provide additional insight for soft sensor building. In the previous findings, it was shown that processes possess the highest variance with respect to biomass trends and endpoint values at an induction ratio of 0.9 (Figure 1). Thus, the PCA scores (PC 1 versus PC 2) for the nine CPP combinations carried out at this ratio are presented in Figure 2C. The different shapes represent different progressions in the fluorescence spectra, caused by the respective CPP combinations. For PC 1, no major difference was found between the fermentations. All scores followed the same course, while the second PC displayed two score groups with contrary trends. All settings at µ = 0.10 displayed unique trajectories (black, orange and turquoise). The courses of the red (30°C and µ = 0.15) and green (30°C and µ = 0.20) trajectories follow the shape of the turquoise one to some extent, but not markedly. It is not surprising that the scores of the blue (34°C and µ = 0.15) and brown (37°C and µ = 0.20) CPP settings are almost identical, since their biomass trends and endpoints (endpoints at 25.2 and 24.0 g/L) are also very similar. Shapes also matching these two are observed for the pink (34°C and µ = 0.20) and grey (37°C and µ = 0.15) scores. Due to comparable process behaviors with respect to the biomass trajectory, similar PCA trends are indicated and their locations in the score plot confirmed these findings. The pink trend (endpoint at 29.3 g/L) is located above the identical blue and brown trends, while the grey trend (endpoint at 21.6 g/L) is below them. This also reflects the biomass concentrations. It can be concluded that different CPP settings lead to varying 2D‐fluorescence spectra. By decomposing and investigating these spectra, conclusions about their progress can be made. All these findings strongly suggest that valuable process information is present in the 2D‐fluorescence data.

### 3.3 Comparison of the input variables for soft sensor development

**Figure 3:**
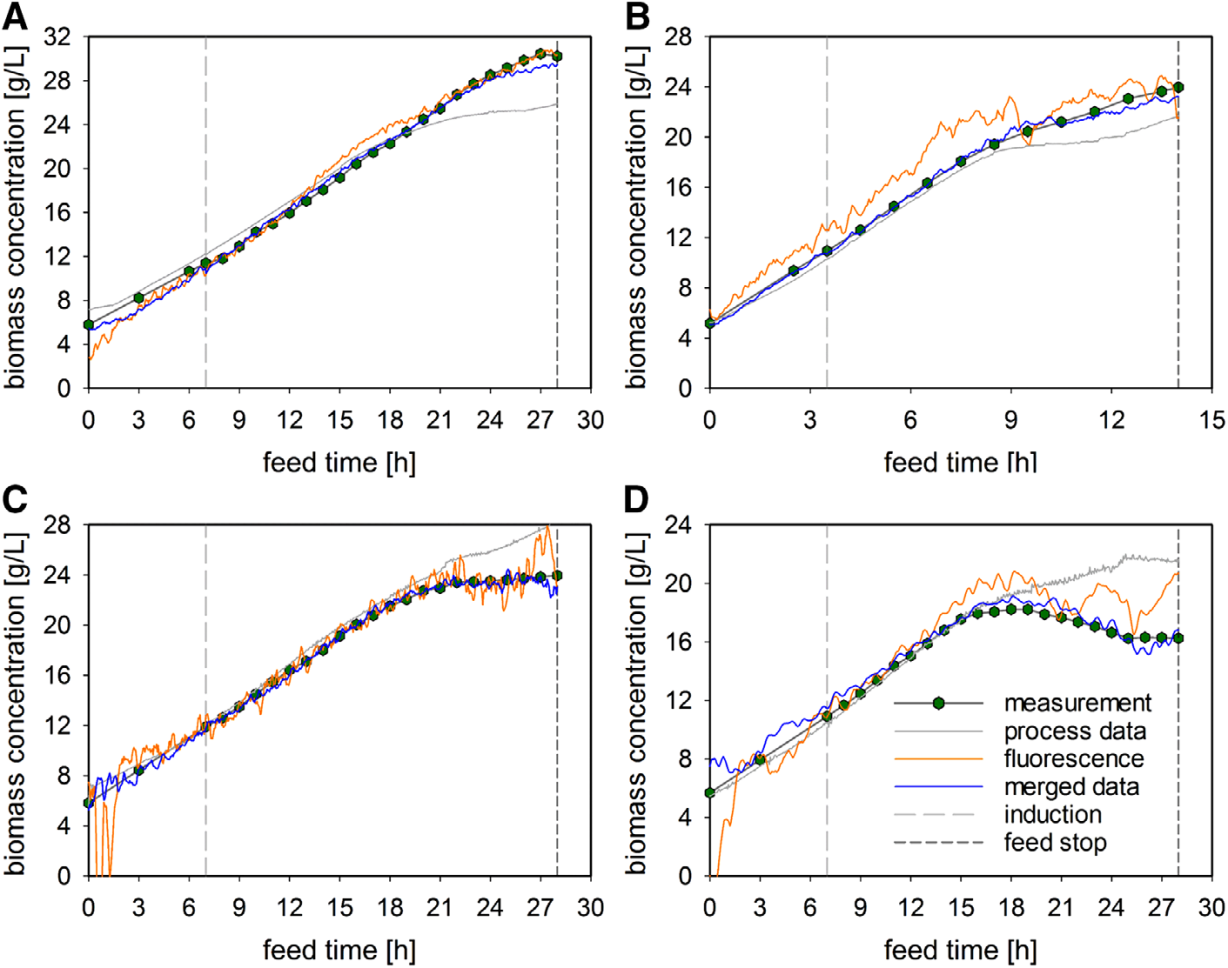
Performance comparison for the different inputs to the model. The biomass trends of four fermentations, the respective time point of induction and the feed stop are shown. (A) 30°C, µ = 0.10 and induction ratio = 0.5; (B) 37°C, µ = 0.20 and induction ratio = 0.9; (C) 34°C, µ = 0.10 and induction ratio = 0.9; (D) 37°C, µ = 0.10 and induction ratio = 0.9. The estimations of the established models using the three different inputs (process data (grey), 2D‐fluorescence data (orange) and merged data (blue)) applied to the LoBo‐CV, are shown

Subsequently, after the exploratory data analysis of the on‐line data, the optimal data set for model‐building was tested using three different types of input. The performances of soft sensors using only process data, using only 2D‐fluorescence data and using merged input data (both types) were considered. For the decision‐making, the best performance with LoBo‐CV (internal validation) was investigated and presented (Figure 3). Four fermentations from the investigated space and the respective model performances are shown. To allow for meaningful comparison and statements, different biomass trends are presented. Two fermentations with consistently increasing concentrations (Figure 3A and B), one reaching a plateau (Figure 3C) and one with a decreasing concentration (Figure 3D), were chosen. The performance criteria (*R* ^2^, RMSE, and the percentage error) for the three established models, are provided in Table 1. The estimation of the soft sensor using solely process data (grey) always, without exception, underestimates or overestimates the measured values, with an RMSE of 1.42 g/L (7%). Using 2D‐fluorescence data (orange) as input to the model led to visually more reliable models, even though the RMSE was higher, reaching 2.04 g/L (10.0%). This occurs due to peaks and fluctuations in the estimations, which are not observed for the model developed using the process data. Only the established soft sensor using both kinds of data (blue) can accurately estimate every trend, resulting in an RMSE of 0.99 g/L (4.9%). This demonstrates that both data sets possess relevant and complementary information for building a robust model and further highlights the importance and advantage of this advanced sensor type.

**Table 1:** Performance results of the developed models with respect to *R* ^2^ and RMSE (both rounded to two decimal places), indicated as concentrations, and the percentage error (rounded to one decimal place), are presented. The different inputs for building the model are indicated. The results for the external validation (test sets #1 and #2) are only given for the final model (using merged data)

	*R* ^2^	RMSE (g/L)	Error (%)
	Process data/fluorescence/merged	Process data/fluorescence/merged	Process data/fluorescence/merged
Training	0.97 / 0.99 / 0.99	1.15 / 0.74 / 0.45	5.7 / 3.7 / 2.2
5x‐CV	0.97 / 0.97 / 0.99	1.28 / 1.20 / 0.58	6.3 / 5.9 / 2.9
LoBo‐CV	0.96 / 0.92 / 0.98	1.42 / 2.04 / 0.99	7.0 / 10.0 / 4.9
Test #1	‐ / ‐ / 0.66	‐ / ‐ / 2.62	‐ / ‐ / 13.8
Test #2	‐ / ‐ / 0.98	‐ / ‐ / 0.69	‐ / ‐ / 3.8

### 3.4 Soft sensor performance of the final model

**Figure 4:**
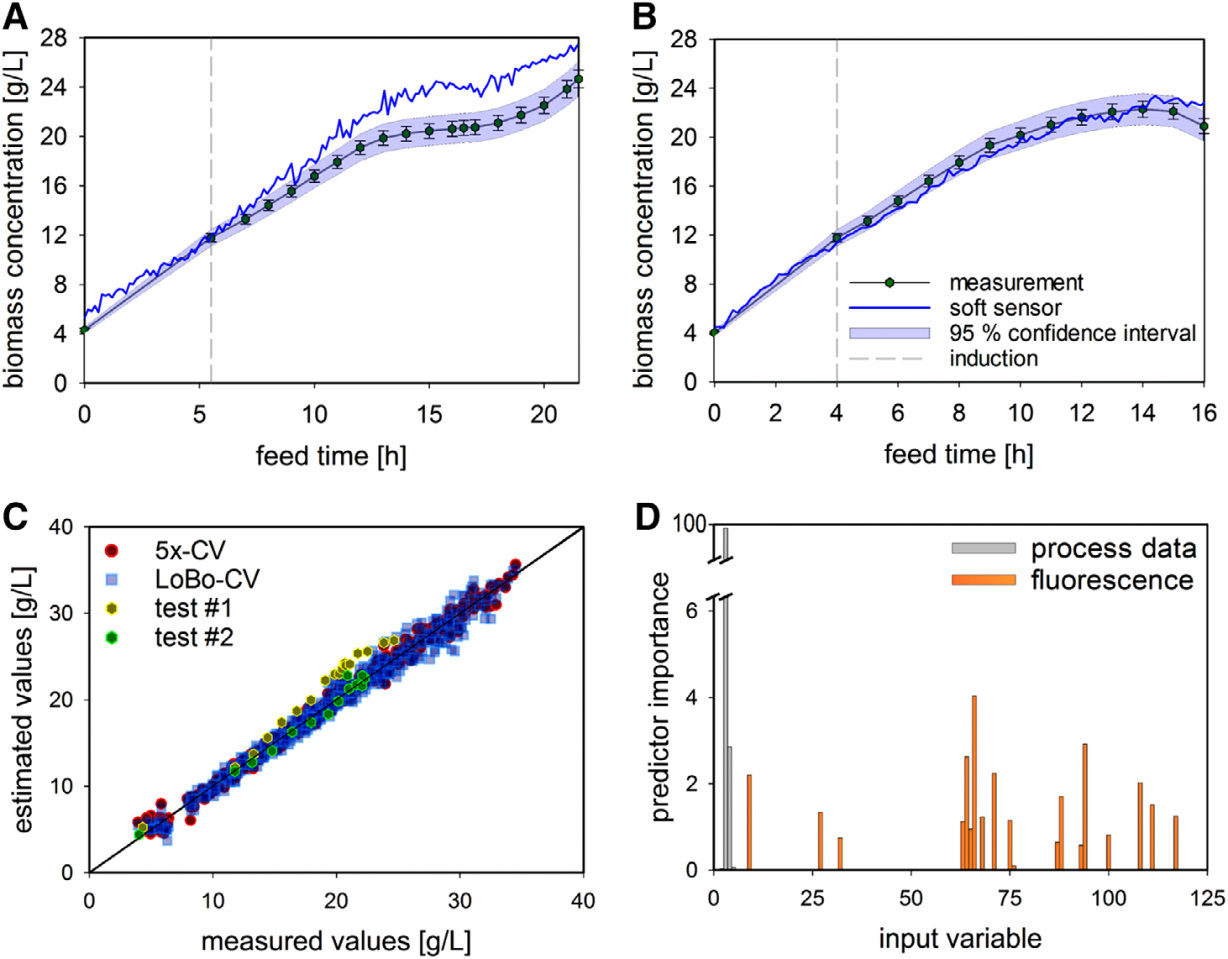
Performance of the final model on the external validation data set. (A–B) The biomass trends for the first (A) and the second (B) test set ± SD including the 95% CI and the estimation from the developed soft sensor, are presented. (C) Scatter plot of the model performances on the 5x‐CV (red dots), the LoBo‐CV (blue squares) and the two test fermentations (yellow and green dots), are shown. (D) The VIP of the process (grey) and 2D‐fluorescence data (orange) derived from the MARS algorithm

Based on the results shown in Figure 3, the soft sensor developed using the merged data set was chosen as the final model. To evaluate the quality of the established soft sensor, its performance on the test set, consisting of two fermentations with (partly) different CPP settings, was considered, that is, external validation was performed. The model's estimation of biomass concentration for the test fermentation, which was executed using three different CPP settings, shows an overestimation after the first half of the process (Figure 4A) with respect to the off‐line measured concentrations. Although the general shape of the trajectory is reproduced, the endpoint is still overestimated at 27 g/L, rather than the analytically measured 24.7 g/L. The estimation of the process with only one different CPP is able to follow the off‐line trend and results in a more satisfying endpoint value of 22.7 g/L, rather than 20.9 g/L (Figure 4B). The higher deviation from the measured values for the first test process is also visible in the scatter plot (Figure 4C), while the results for the second process are located within the error magnitude of the two internal cross‐validations. Since the training error is negligibly small, at 0.45 g/L (2.2%), these results are not displayed in the scatter plot, for greater clarity. The remaining variance in the test sets might result from a factor that is not considered in the model input or from a function of included factors interpreted in an insufficient way by the model. To evaluate which input variables are important for the estimation, the VIP was determined for the five process parameters as well as for the 120 ex/em pairs. As shown in Figure 4D, only a few inputs are important for building the model. These were therefore retained in the backward deletion phase of model‐building and included in the algorithm. The list of all variables with VIP scores above zero, in descending order, is presented in Supporting Information Table S2. The highest importance for the process parameters was given to the accumulated feed (scoring the maximum value of 100) and the accumulated inductor (scoring 2.8). For the 2D‐fluorescence data, only 19 of the 120 available variables were taken into account in model‐building. The chosen variables are mostly collinear, due to the fact that they are neighbors (ex/em ± 20 nm). These collinear ex/em pairs do not carry extra information but are still included in the model for noise reduction and enhanced robustness. This finally results in only two important ex/em pairs, namely, ex450/em530 (scoring 2.9) and ex370/em470 (scoring 4). These are identical to the two ex/em pairs determined via PARAFAC. The final model performance with respect to the RMSE of the 5x‐CV is fairly small, at 0.58 g/L (2.9%), and the LoBo‐CV also displays good accuracy with an RMSE of 0.99 g/L (4.9%). The results for the external validation show an RMSE score of 2.62 g/L (13.8%) with three altered CPPs and 0.69 g/L (3.8%) with one altered CPP, highlighting the estimation qualities of the established soft sensor. This is in good accordance with the off‐line measurement used as the reference, where an SD of 3.41% was observed. The performance of the final model on the test set is presented in Table 1.

## 4 DISCUSSION

The impact of the CPP settings on the biomass is shown in Figure 1. The different concentrations and endpoints result from diverse metabolic burdens, for example, recombinant protein production, stressing the cells. With slow SGR settings, more resources are available for protein synthesis. When the SGR increases, cells focus more on their own growth and neglect protein production. As a result, fewer product is present and stress levels are lower. This results in higher biomass concentrations, even though the other CPP settings, except the SGR, stay the same. The product formation, metabolism and cellular stress levels also increase with higher temperature settings, again leading to decreased biomass values. The maximum impact is seen when considering the opposing corners of the investigated design space, resulting in more than 50% difference, at 16.3 and 34.5 g/L.

The insights into the fluorescence data via PCA and PARAFAC (Figure 2) strongly support the assumption that, in fact, it is possible to monitor intracellular fluorescent substrates, especially the ex/em wavelengths of the two chemical compounds identified via PARAFAC are similar to those from flavins (riboflavin, flavin mononucleotide and flavin adenine dinucleotide) for factor 1 30 and nicotinamide adenine dinucleotide phosphate for factor 2 31. These molecules are directly linked to cell physiology and important metabolic pathways. Flavins are overproduced during exponential growth and act as electron carriers 32. Similarly, nicotinamide adenine dinucleotide phosphate is a major component of the electron transfer chain 33. It is conceivable that different DoE settings result in diverse concentrations and consumption rates. Since metabolic activities are temperature‐dependent, it is indicated that these changes and deviations in cell physiology caused by different CPP combinations are measured by the 2D‐fluorescence probe. This is also hinted at by the two observable score groups of PC 2. They are caused by two different observed ex/em wavelength clusters in the loadings of PC 2. One group contained a cluster with wavelengths ex450/em510‐550 and the second group contained a cluster with wavelengths ex510‐530/em550‐590. These show different trends over the fermentation and cause the opposing trend in the PCA score plot. However, more investigation into the cell is required in the future, for example, taking cell lysis into account or deliberately provoking metabolic shifts and measuring the response in the fluorescence spectra.

In addition, the added value and advantage of using a 2D‐fluorescence probe for on‐line biomass estimation is demonstrated across CPP settings, and also for fermentations with altered CPP settings. With the process data alone, only discrete and accumulating/rising values are introduced into the model. Thus, fermentations with steady or decreasing concentrations are especially difficult to estimate, as previously shown (Figure 3). Using 2D‐fluorescence in addition allowed the cell physiology and metabolism to be examined and the potential underlying chemical compounds to be identified.

The test fermentation with three altered process settings (Figure 4A) led to completely new metabolic patterns for which the MARS model was not trained, and therefore resulted in a high residual value. To overcome these boundaries, other CPP levels could be considered and additional sensors could be utilized. However, these approaches would need to be accompanied by several additional experiments. To avoid this time‐consuming step most simply, a mechanistic part (white box) can be taken into account to describe this missing term. This exploitation of both model advantages is called hybrid modeling, and has already been reported elsewhere 34. Potentially, with this added value, more challenging processes can also be monitored on‐line in the future, such as the so‐called intensified DoE. Hence, through intra‐experimental set‐point changes, the dynamics of the specified design space can be captured 35.

MARS proved to be a suitable algorithm for soft sensor development (Figure 4). Its characteristics, for example, its ability to handle nonlinearity and multicollinearity, make it an ideal candidate for working with this complex input data and creating meaningful models. Its VIP also determined the importance of two particular ex/em pairs for accurate biomass estimation. Moreover, these were identical to the factors identified by PARAFAC. As discussed above, these wavelengths probably represent chemical compounds that are representative of the current biomass state. It is comprehensible that the highest VIP for biomass estimation is possessed by the amount of added feed medium (controlling the SGR). However, the two ex/em variables seem to be responsible for fine‐tuning the precise biomass estimation by the soft sensor, taking the various metabolic burdens into account. This enables precise on‐line monitoring of the biomass with real‐time availability of the current value, which can be exploited in the QbD concept. All these findings demonstrate that the established soft sensor is a valuable PAT tool.

The study did not contain experiments using animals or human subjects.

## CONFLICT OF INTEREST

The authors have declared no conflict of interest.

## Supporting information

Supporting Information
