# Supplementary material for: Soft sensor based on 2D‐fluorescence and process data enabling real‐time estimation of biomass in Escherichia coli cultivations
Source: Eng Life Sci. 2019 Nov 11;20(1-2):26–35. doi: 10.1002/elsc.201900076 (PMC6999058; doi:10.1002/elsc.201900076)
Supplement: Supplementary file 1 — Supporting Information [file ELSC-20-26-s001.pdf]

Supporting Information

All the critical process parameter settings, presented in the Materials & Methods section of the main manuscript (2.2), investigated in the design space for model building and internal validation, as well as the settings for the external validation (test sets), are presented in Table S1.

Table S1. All investigated CPP combinations of the design space, namely, specific growth rate, induction strength and cultivation temperature (each with three levels) used as training are listed as well as the settings for the test set. If more than one fermentation was performed, the number of repetitions is indicated.

CPP setting	temperature [°C]	specific growth rate [h^{-1}]	induction ratio [µmol IPTG/g cell dry mass]
1	30	0.1	0.2
2	30	0.15	0.2
3	30	0.2	0.2
4	34	0.1	0.2
5	34	0.15	0.2
6	34	0.2	0.2
7	37	0.1	0.2
8	37	0.15	0.2
9	37	0.2	0.2
10 (N = 2)	30	0.1	0.5
11	30	0.15	0.5
12 (N = 2)	30	0.2	0.5
13	34	0.1	0.5
14	34	0.15	0.5
15	34	0.2	0.5
16	37	0.1	0.5
17	37	0.15	0.5
18	37	0.2	0.5
19	30	0.1	0.9
20	30	0.15	0.9
21	30	0.2	0.9
22	34	0.1	0.9
23	34	0.15	0.9
24	34	0.2	0.9
25 (N = 5)	37	0.1	0.9
26	37	0.15	0.9
27	37	0.2	0.9
28 (test set #1)	35	0.13	0.75
29 (test set #2)	37	0.17	0.9

The relative importance of each input variable for the multivariate adaptive regression spline model-building, presented in the Results section of the main manuscript (3.4) in Fig. 4D, is listed in descending order in Table S2. Only variables that were used for building the final model are listed, i.e., scoring a VIP above zero.

Table S2. List of all VIP scores (above zero) of the final model in descending order.

rank	input variable	VIP score
1	accumulated feed	100.0
2	ex370/em470	4.0
3	ex450/em530	2.9
4	accumulated inductor	2.8
5	ex350/em470	2.6
6	ex370/em410	2.2
7	ex270/em370	2.2
8	ex450/em550	2.0
9	ex390/em570	1.7
10	ex470/em510	1.5
11	ex290/em450	1.3
12	ex490/em550	1.3
13	ex350/em550	1.2
14	ex370/em490	1.2
15	ex350/em450	1.1
16	ex350/em490	1.0
17	ex430/em510	0.8
18	accumulated base	0.8
19	ex290/em550	0.8
20	ex390/em550	0.6
21	ex410/em510	0.6
22	temperature	0.4
23	ex370/em510	0.1

The reactor volumes of the presented *E. coli* fed-batch cultivations in the Materials & Methods section of the main manuscript (2.1) utilizing an exponential feeding rate profile are presented (Fig. S1). The batch medium was calculated to produce 22.5 g biomass. This value was used as specific growth rate setpoint to calculate the exponential feeding strategy to constantly provide the respective set specific growth rate during the whole feeding phase. Volume differences are observable due to batch-to-batch variations and different base consumption patterns due to the varying critical process parameter combinations.

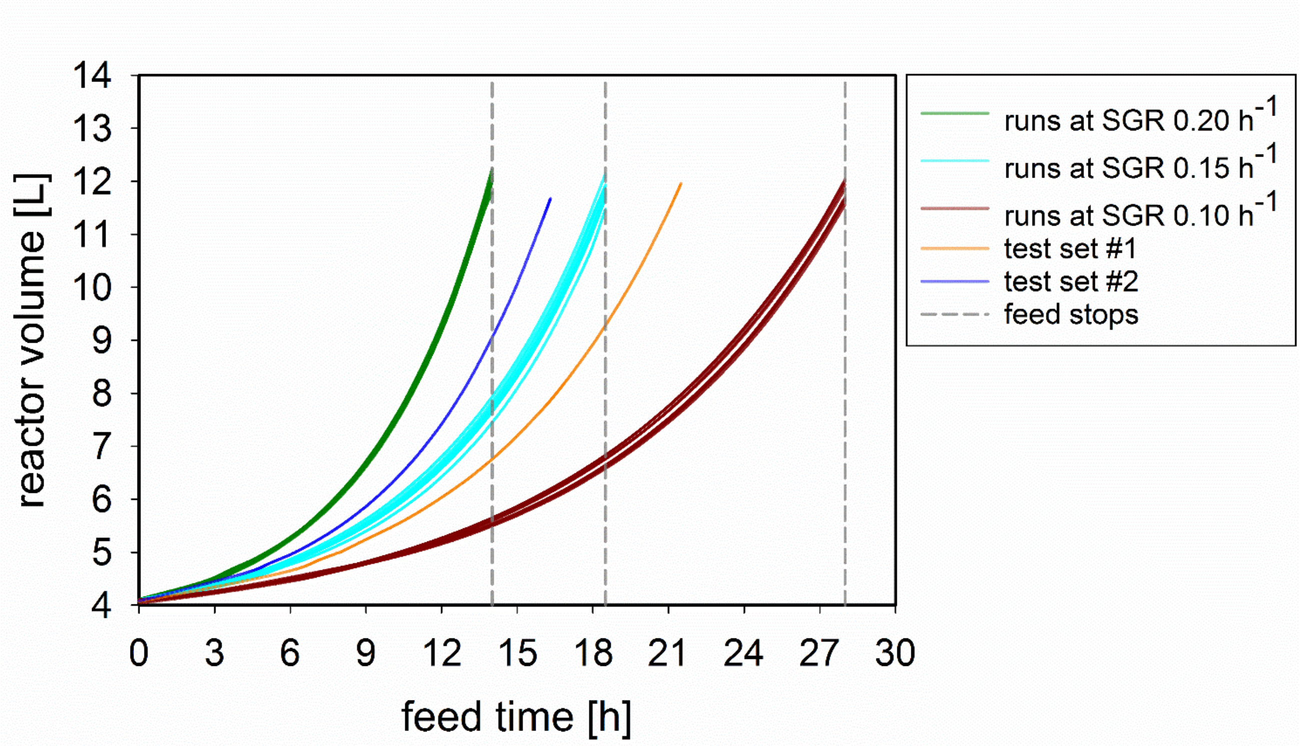

Figure S1. Reactor volumes of all DoE fed-batch fermentations applying an exponential feeding strategy. The reactor volumes as a function of the SGR, slow (dark red), medium (cyan) and fast (dark green), are shown for every CPP setting of the DoE study. The time of the respective feed stop (14, 18.5 and 28 h) is indicated (dashed grey lines). Test set #1 (orange) and test set#2 (blue) are displayed, without the respective feed stops (21.5 and 16 h).

The applied workflow of the soft sensor development, using MATLAB 2016b and additionally the freely available toolbox packages, described in the Materials & Methods section of the main manuscript (2.2), is presented in the simplified form of a graphical overview (Fig. S2).

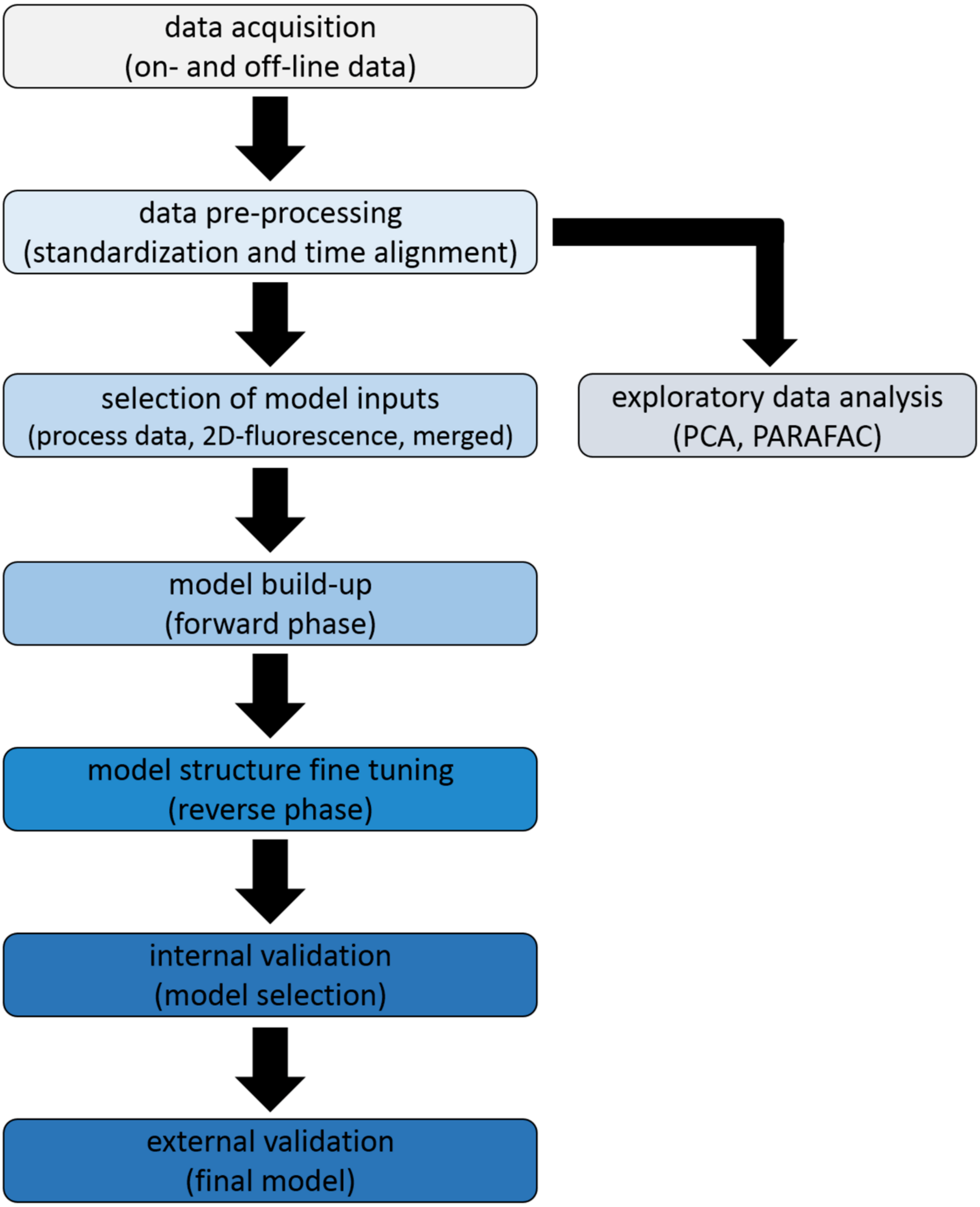

Figure S2. The schematic stepwise workflow of the model development.

The MARS algorithm used for building the soft sensor is considered as a flexible tool for regression modeling. Its strengths, compared to other algorithms used for regression modeling, are higher flexibility to capture relationships and interactions between input variables. This makes it an ideal candidate for handling high dimensional data with additive and (multi)collinearity characteristics, as it is the case for the 2D-fluorescence data. This dynamic adaptability is enabled by the selection of a subset of local variables. These are depending on distinctive conditions to fit the target value.

In general, the MARS algorithm can be seen as a specialization of a general multivariate regression and an expansion to spline basis functions, in which the number of basis functions is automatically limited by the number of inputs. The MARS algorithm builds hierarchical models in a two-step procedure; forward and backward. Starting with a set of basis functions, by stepwise selection a subset of these is chosen, which are suitable for modeling the target variable.

In the first phase (forward selection procedure), the model comprises only the intercept term and more basis functions are added iteratively to consecutively reduce the training error. This phase is executed until any of the conditions to stop are met, e.g., the number of coefficients equals the number of observations, new basis functions do not change the R^2 above the set threshold or the R^2 reached 1. The result of the forward phase is a large model overfitting the data. This general structure of the main model is given in Eq. 1. The estimated value ($\hat{y}$) of the model, the intercept *(b)*, a basis function *(BF)* and its respective covariate vector (v) for the number of used functions (i) is given.

$$\hat{y} = b + v_{(1)} * BF_{(1)} + \ldots + v_{(i)} * BF_{(i)} \qquad (1)$$

Each truncated cubic basis function comprises one of the input variables and three respective knot locations (a central knot, a lower and an upper side knot) to handle the local conditions. After completing the first phase, the, in this study, developed overfitting model comprised 65 of these basis functions.

To optimize the established overfitting model, the second phase (backward deletion procedure) is executed to simplify and generalize the model. This is done by stepwise deleting the least important basis function (smallest reduction of the training error) until the model again only consists of the

intercept term. For every reduced model, the generalized cross-validation (GCV) is determined (Eq. 2). The reduced model for which the lowest GCV, with optimal performance on validation data, is obtained, is selected as the final model. The GCV is calculated using the models mean squared error (MSE_{train}), the number of observations (N) and the effective number of parameters (n_p) in the model.

$$GCV = MSE_{train} / \left(1 - \frac{n\_p}{N}\right)^2 \tag{2}$$

The number of initially used input variables was reduced from 125 to 23 (Table S2), which were applied in the basis functions. The number of basis functions remaining in the final model is pruned to 42, including the intercept term. The number of final basis functions exceeds the number of finally used input variables, which means that some input variables were assigned multiple times to the remaining basis functions. The obtained final model was applied to the test set fermentations (external validation) to demonstrate its performance on new data, which had not been used for validation.

The input variables used in the final MARS model (see Table S2) are shown in Fig. S3. The fed-batch fermentation performed at CPP setting 25 (see Table S1) is shown as an example to demonstrate their respective trajectory during the fed-batch fermentation. The on-line available process variables in Fig. S3A present an exponential (feed, base and inductor, according to the feeding profile) or constant (temperature, setpoint at 37°C) trend. Otherwise the ex/em wavelength pairs in Fig. S3B consist of diverse shapes, e.g., (multi)collinear, nonlinear and constant trends. For an easier comparison and a simplified visual inspection of the 2D-fluorescence data, each ex/em wavelength pair was scaled from zero to one. The two ex/em wavelength pairs identified by PARAFAC and MARS are highlighted.

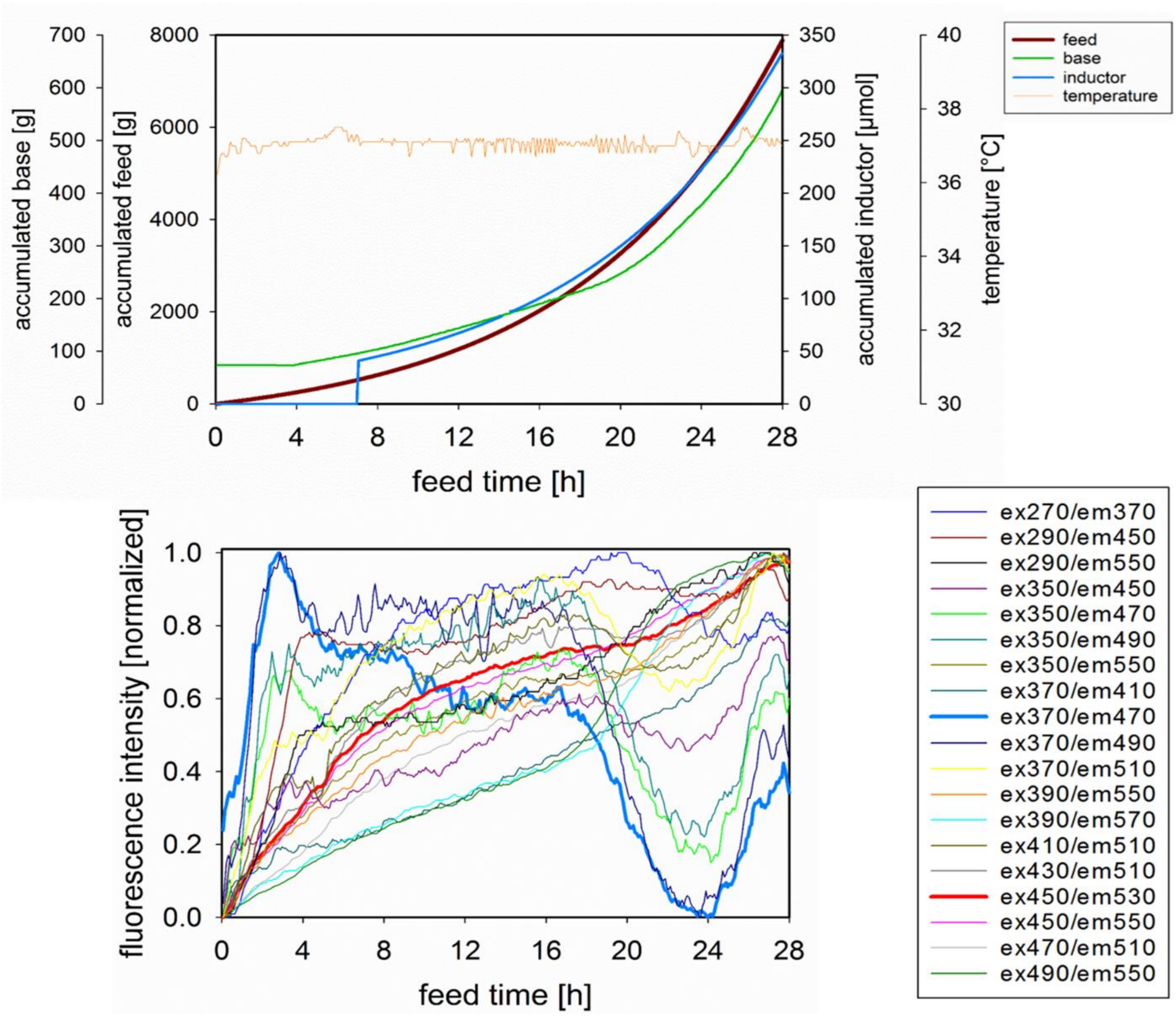

Figure S3. Trajectories of the input variables over the whole fed-batch fermentation at CPP setting 25. A: The on-line available process variables used as input to the MARS model, namely, the feed (dark red), base (green), inductor (blue) and temperature (orange), are displayed. B: The 19 ex/em wavelength pairs used as input to the MARS model are displayed. Each ex/em wavelength pair was scaled from zero to one. The two ex/em wavelength pairs, identified both by PARAFAC and MARS, are highlighted (ex370/em470 in blue and ex450/em530 in red).
